# Severity of COVID-19 Infection Using Chest Computed Tomography Severity Score Index Among Vaccinated and Unvaccinated COVID-19-Positive Healthcare Workers: An Analytical Cross-Sectional Study

**DOI:** 10.7759/cureus.22087

**Published:** 2022-02-10

**Authors:** Bukke Ravindra Naik, Sakalecha Anil Kumar, N Rachegowda, L Yashas Ullas, RB Revanth, Nikhilendra Reddy Venkata Sai Aluru

**Affiliations:** 1 Radiology, Sri Devaraj Urs Medical College, Karnataka, IND; 2 Department of Radio-Diagnosis, Sri Devaraj Urs Medical College, Kolar, IND

**Keywords:** disease severity, prevalence, covid-19 infection, healthcare workers, post-vaccination

## Abstract

Introduction: Coronavirus disease 2019 (COVID-19) vaccines protect against severe illness. However, data on post-vaccination COVID-19 breakthrough infections are limited.

Methods: An analytical cross-sectional study was conducted from May 2021 to July 2021 among 2043 COVID-19-positive healthcare workers who were divided into a vaccinated group (n=1010) and an unvaccinated group (n=1033). A pre-tested questionnaire was circulated among the healthcare workers using Google Forms. Chest computed tomography (CT) severity score was the primary outcome variable analyzed using coGuide.

Results: The average age of the study population was less than 45 years in both groups (43.05 ± 13.02 years). Most respondents (62%) were males. Hypertension (39%) and diabetes (33%) were the most common underlying diseases. Significant differences in age and cardiac disease were observed between the two groups (*p* = 0.07 and *p* <0.001, respectively). However, the difference was insignificant (*p* >0.05) for gender, hypertension, and diabetes. Most unvaccinated respondents had an increased CT severity score, and the difference between the studies groups was significant (*p* <0.001). Of the 1,010 vaccinated individuals, 382 (37.82%) received the only first vaccination dose, and 628 (62.18%) received both doses. The CT severity score decreased after receiving both vaccination doses, and the difference between CT severity score and vaccination status was significant (*p* <0.001).

Conclusion: COVID-19 was mild in the vaccinated group. Chest CT severity score index can be considered an efficient tool in predicting prognosis and monitoring disease in patients with COVID-19 in India.

## Introduction

Since its emergence in December 2019, the severe acute respiratory syndrome coronavirus 2 (SARS-CoV-2) pandemic has caused high morbidity and mortality, with new variants rapidly spreading. Vaccines have been developed to prevent coronavirus disease 2019 (COVID-19) with unprecedented speed [1,2].

The first vaccines against SARS-CoV-2 have now received emergency use authorization by the US Food and Drug Administration, European Medicines Agency, and national authorities in China, India, the UK, and Russia and are rapidly being distributed in more than 30 countries since the end of 2020 [3]. In India, the vaccination program for COVID-19 started on January 16, 2021, operating 3,006 vaccination centers. Initially, frontline health and social care workers and those over 60 years of age were prioritized for vaccination. Vaccines, such as Covishield and Covaxin, in two doses, have been introduced in India. Ongoing assessment of the effectiveness of different vaccines across different subgroups is critical due to vaccination shortages and increasing infections.

Real-world studies from Israel and England have reported 92% (95% confidence interval (CI), 88% to 95%) effectiveness against symptomatic polymerase chain reaction (PCR)-confirmed infection ≥seven days after the second Pfizer-BioNTech dose [4,5]. Recent studies in the US have reported 80% to 90% vaccine efficacy among healthcare workers, first responders, and other essential and frontline workers [6,7]. Chest computed tomography (CT) scans with 95% sensitivity are considered the primary diagnostic tool in detecting COVID-19 [8]. Recently, the Dutch Radiological Society created the COVID-19 Reporting and Data System (CO-RADS) upon suspicion for COVID-19 pneumonia in chest CT images [9,10].

Though COVID-19 vaccines protect against severe illness, post-vaccination SARS-CoV-2 infections are expected, as 100% protection is not offered by these vaccines (breakthrough infections) [11]. The current literature is deficient in explaining the correlation of chest CT severity scores in patients who have been vaccinated for COVID-19 and have tested positive on reverse transcription (RT)-PCR after vaccination. With this background, this study was designed to assess the severity of COVID-19 among vaccinated and unvaccinated COVID-19-positive healthcare workers using chest CT severity scores. This study was designed to assess the severity of COVID-19 among vaccinated and unvaccinated COVID-19-positive healthcare workers of Karnataka.

## Materials and methods

Study design

An analytical cross-sectional study was conducted using an online survey among 2,043 healthcare workers of Karnataka from May 2021 to July 2021. Ethical permission was obtained from the Institutional Review Board of the concerned tertiary care center (reference number: SDUMC/KLR/IEC/243/2021-22). Written informed consent was obtained from all participants. The information present in the questionnaire was explained to the participants in detail.

Sample size calculation

The sample size was calculated assuming that the proportion of patients with severe COVID-19 with chest CT severity score was 16.7% in the vaccinated group and 29.9% in the unvaccinated group according to the pilot study. The other parameters considered for sample size calculation were 90% power and 95% confidence level. The required sample size according to the aforementioned calculation was 210 in each group. To account for a nonparticipation rate of approximately 30%, another 63 subjects were added to the sample size. Hence, the final required sample size would be 273 in each group. According to the availability of cases, 1,010 and 1,033 in the vaccinated and unvaccinated groups, respectively, were considered as per convenience sampling method for the feasibility of the study.

Inclusion criteria

Patients infected with COVID-19 after vaccination who tested positive on RT-PCR and have undergone high-resolution CT (HRCT) of the thorax and patients infected with COVID-19 who were unvaccinated, tested positive on RT-PCR, and have undergone HRCT of the thorax.

Exclusion criteria

Patients who have tested positive on RT-PCR and have not undergone HRCT of the thorax and patients who have tested negative on RT-PCR and have undergone HRCT of the thorax.

Data collection

The questionnaire was circulated among healthcare workers of Karnataka using Google Forms. The questions were developed to collect data regarding vaccination status, COVID-19 positivity after vaccination, and CT severity score. Few questions were used from a previous retrospective study on COVID-19-positive healthcare workers post-vaccination by Patil et al. [12], in Mumbai Maharashtra. A pilot study involving 30 healthcare workers was conducted to test the questionnaire.

The Dutch Radiological Society created the CO-RADS, which standardizes the assessment scheme and simplifies reporting using a five-point scale of suspicion for COVID-19 pneumonia on chest CT images.

The CO-RADS categories used for CT scoring are summarized as follows Table 1 [9]:

**Table 1:** Summary of CO-RADS categories used for CT scoring CO-RADS: COVID-19 Reporting and Data System; SARS-CoV-2: severe acute respiratory syndrome coronavirus 2; COVID-19: coronavirus disease 2019; RT-PCR: reverse transcription polymerase chain reaction

CO-RADS score	Level of suspicion	Findings
CO-RADS 0	Not interpretable	Scan technically insufficient for assigning a score
CO-RADS 1	Very low	Normal or noninfectious
CO-RADS 2	Low	Typical for other infections but not COVID-19
CO-RADS 3	Equivocal/unsure	Features compatible with not only COVID-19 but also other diseases
CO-RADS 4	High	Suspicious of COVID-19
CO-RADS 5	Very high	Typical for COVID-19
CO-RADS 6	Proven case	Positive for SARS-CoV-2 on RT-PCR

Each patient was categorized as CO-RADS 0, 1, 2, 3, 4, 5, or 6 based on the aforementioned findings.

The CT severity score index [13,14] is a scoring system used to assess lung changes and involvement caused by COVID-19 based on the estimation of pulmonary involvement. Each of the five lung lobes (three right and two left lobes) has been visually scored and given a score from 1 to 5 (Table 2, 3).

**Table 2:** The CT severity score index

Percentage of involvement (single lobe)	Score
0%–5%	1
5%–25%	2
25%–50%	3
50%–75%	4
75%–100%	5

**Table 3:** Calculation of CT severity score

CT severity	Score
Mild	<8
Moderate	9–15
Severe	>15
Total score	~25

Study variables

CT severity score was the primary outcome variable.

Statistical methods

Descriptive data were presented as means ± standard deviations for age and as frequency and proportions for gender and comorbidities. Age and comorbidities were compared across the groups using an independent t-test, and CT severity scores were compared among the groups using the chi-square test. P-values of less than 0.05 were considered statistically significant. Data were analyzed using coGuide software (V.1.03) [15].

## Results

All 2,043 subjects were considered for final analysis.

Of the 2,043 healthcare workers, 49.4% received vaccination and 50.6% were unvaccinated. The ratio of vaccinated and unvaccinated individuals in the study population was almost equal.

The mean age was 43.05 ± 13.02 years in the vaccinated group and 42.05 ± 13.15 years in the unvaccinated group. Most respondents (62%) were males in both groups. Hypertension (39%) and diabetes (33%) were the most common underlying diseases in both groups. Moreover, 98 (9.70%) healthcare workers in the vaccinated group had cardiac disease, whereas only 13 (1.26%) healthcare workers in the unvaccinated group had cardiac disease. However, significant differences in age and the prevalence of cardiac disease were observed between the two groups (p = 0.07 and p <0.001, respectively), and the differences in sex ratio and comorbidities, such as hypertension and diabetes, between the two groups were insignificant (p >0.05) (Table 4).

**Table 4:** Comparison of baseline parameters between the study groups (N = 2043). *Independent sample t-test,  † Chi-square test. COPD: chronic obstructive pulmonary disease

Parameter	Study group		P-value
	Vaccinated (N = 1010)	Unvaccinated (N = 1033)	
Age	43.05 ± 13.02	42 ± 13.15	0.070*
Gender			
Male	621 (61.49%)	645 (62.44%)	0.657†
Female	389 (38.51%)	388 (37.56%)	
Comorbidities			
History of diabetes			
Yes	393 (38.91%)	374 (36.21%)	0.207†
No	617 (61.09%)	659 (63.79%)	
History of hypertension			
Yes	338 (33.47%)	328 (31.75%)	0.409†
No	672 (66.53%)	705 (68.25%)	
History of asthma/COPD			
Yes	76 (7.52%)	75 (7.26%)	0.819†
No	934 (92.48%)	(2.74%)	
Cardiac disease			
Yes	98 (9.70%)	13 (1.26%)	<0.001†
No	912 (90.3%)	1020(98.74%)	

Most participants in the unvaccinated group had an increased CT severity score compared with those in the vaccinated group. Of the 1,033 participants in the unvaccinated group, 154 (14.91%) showed severe CT scores, whereas, of the 1,010 individuals in the vaccinated group, only three had severe CT scores. The difference in CT severity score between the study groups was significant (p <0.001) (Table 5).

**Table 5:** Comparison of clinical parameters between the study groups (N = 2043). *No statistical test was applied due to 0 subjects in the cells, † Chi-square test. COVID-19: coronavirus disease 2019; RT-PCR: reverse transcription polymerase chain reaction

Parameter	Study group		P-value
	Vaccinated (N = 1010)	Unvaccinated (N = 1033)	
RT-PCR test for COVID-19 infection			
Positive	1010 (100%)	1,033 (100%)	*
CT severity score			
Normal study	514 (50.89%)	197 (19.07%)	<0.001†
Mild	382 (37.82%)	416 (40.27%)	
Moderate	111 (10.99%)	266(25.75%)	
Severe	3 (0.3%)	154 (14.91%)	

 Of the 1,010 vaccinated individuals, 382 (37.82%) received only the first dose and 628 (62.18%) received both vaccination doses (Table 6).

**Table 6:** Summary of vaccination status (N = 1010).

Vaccination status	Percentages
First dose	382 (37.82%)
Second dose	628 (62.18%)

The CT severity score was reduced after receiving both vaccination doses. Only 10 (1.59%) of the 628 individuals who received both vaccination doses showed moderate severity scores after receiving both vaccination doses compared with those who received only a single dose of vaccination where 101 (26.44%) had moderate scores after receiving the first dose. The difference in CT severity score and vaccination status was significant (p <0.001) (Table 7).

**Table 7:** Comparison of CT severity score in terms of vaccination status (N = 1010).

CT severity score	Vaccination status		Chi-square	P-value
	First dose completed (N = 382)	Second dose completed (N = 628)		
Normal study	140 (36.65%)	374 (59.55%)	158.168	<0.001
Mild	140 (36.65%)	242 (38.54%)		
Moderate	101 (26.44%)	10 (1.59%)		
Severe	1 (0.26%)	2 (0.32%)		

## Discussion

To the best of the author’s knowledge, this is the first study that has assessed the severity of COVID-19 using CT severity scores among vaccinated and unvaccinated COVID-19-positive healthcare workers of Karnataka. The CO-RADS classification developed by the Dutch Radiological Society is a simple interpretation method with a high inspection accuracy and matching rate. This is one of the few studies that verified the effectiveness of the CO-RADS in assessing the severity of COVID-19. The difference in chest CT severity scores between the study groups was significant (p <0.001) with severe scores observed only in three (0.3%) healthcare workers in the vaccinated group compared with 154 (14.91%) healthcare workers in the unvaccinated group. As data related to breakthrough infections after vaccination for COVID-19 are limited, we included nearby studies related to COVID-19 to compare the findings of this study. The mean age was 43.05 ± 13.02 years in the vaccinated group and 42.05 ± 13.15 years in the unvaccinated group, and the difference in age was statistically significant (p = 0.07). Moustsen-Helms et al. [16] found a similar finding where the mean age was 47 years. Most responders (63%) were males and 37% were female in this study. Al-Mosawe et al. [17] have found that 58.2% were males and 41.8% were females, a finding similar to this study. In this study, 382 (37.82%) healthcare workers received only the first vaccination dose, whereas 628 (62.18%) received both vaccination doses. Indian Council of Medical Research chief Balram Bhargava has reported only 2-4 in 10,000 breakthrough infections (COVID-19 infections post-inoculation) in India so far, which was negligible. Likewise, of the 100,302,745 individuals who received only the first dose of Covishield, 17,145 (0.02%) had breakthrough infections, and among the 15,732,754 who received both Covishield doses, 5,014 reported breakthrough infections [18]. 

In the present study, the CT severity score was reduced after receiving both vaccination doses, and the difference between CT severity score and vaccination status was significant (p <0.001). Hitchings [19], in his case-control study, reported increased odds of symptomatic SARS-CoV-2 infection (odds ratio, 1.69; 95% CI, 1.09-2.64) among vaccinated healthcare workers after receiving the first vaccination dose compared with that among healthcare workers who did not receive the vaccine. Bernal et al. [5] found a 44% lower risk of hospitalization and 51% lower risk of death in symptomatic vaccinated who went on to become asymptomatic cases had a 44% lower risk of hospitalization compared to unvaccinated cases. Amit et al. [20] estimated vaccine effectiveness of 85% from 15-28 days after the first dose, though they also estimated a 45% reduction from days one to 14 which may indicate that those vaccinated had a lower baseline risk date. The differences noted in studies can be due to the differences in the populations analyzed and can also be due to the analytical approach used.

Most vaccinated and unvaccinated individuals had diabetes and hypertension as underlying diseases. Sanyaolu et al. [21], in their systematic review, have found that comorbidities, such as hypertension and diabetes mellitus, are associated with increased COVID-19 complications similar to this study. Most unvaccinated individuals had an increased CT severity score compared with vaccinated individuals, and the difference in CT severity score between the study groups was significant (p <0.001) in this study. Francone et al. [22], in their retrospective study, have found significantly higher CT severity scores in individuals in the critical and severe stages than in those in the mild stage (p <0.0001) and in patients in the late phase than in those in the early phase (p <0.001).

Muruato et al. [23] have developed a reporter assay to protect individuals from SARS-CoV-2. The device monitors the waning of protective neutralizing titers in patients with COVID-19 vaccinated individuals and monitors populations at a high risk of COVID-19. To protect the population, it is imperative that healthcare workers and the general population be vaccinated. Disease transmission among vaccinated populations becomes crucial, and hence, preventive strategies should be implemented continuously. New cases after an outbreak can be declined by the in-time implementation of infection control strategies. The concept behind this study is an addition to the literature on the ongoing pandemic. Experience about infected healthcare workers post-vaccination can alert the general population to undermine the importance of infection control measures, including social distancing, hand sanitization, and wearing of masks, among others.

This study has a few limitations. First, the study design was cross-sectional, which can affect the causal and temporal relationships. The study was conducted on healthcare workers and hence cannot be generalized to the general population. As the data from healthcare workers was collected online the information provided can be subjected to recall biases. Moreover, some important variables, including information on the type of vaccination, hospitalization due to COVID-19, and duration post-vaccination that can affect the efficacy of vaccination, were not recorded. The relationship between CT severity image findings and disease prognosis was not examined. Further research will be needed to validate the findings of this study and to compare the diagnostic performance of the CO-RADS in assisting radiologists and improving their accuracy and efficiency in diagnosing COVID-19. This was not an efficacy study or a randomized controlled trial, and hence, further multicentre studies with such designs covering a large sample of all age groups are recommended.

## Conclusions

COVID-19 infection can occur even after vaccination among healthcare workers as they are at the highest risk of exposure. CT severity scores were mild in the vaccination group compared with those in the un-vaccination group. Chest CT must be considered the primary diagnostic tool in predicting disease severity in patients with COVID-19. Global vaccine coverage and infection control practices, such as wearing a mask, social distancing, wearing appropriate personal protective equipment, and hand sanitization, will help combat the pandemic. In the pandemic period, laboratory testing for COVID-19 should be performed to differentiate COVID-19 symptoms from side effects of the vaccine in the vaccinated population. During subsequent waves of this novel virus transmission, vaccination can decrease mortality, and disease severity. The chances of getting COVID-19 are low after vaccination because the vaccine helps protect the body against the virus and in case of re-infection, the vaccine reduces the severity of COVID-19 and the possibility of hospital admission and death.

## Appendices

The questionnaire consisted of questions related to

a.     What is your age?

b.     What is your gender?

c.     Are you a health care worker?

d.     Do you have any history of diabetes?

e.     Do you have any history of hypertension?

f.      Do you have any history of asthma/COPD?

g.     Any known cardiac disease?

h.     What is your vaccination status?

i.      Any symptoms related to COVID-19 infection?

j.      RT-PCR test status for COVID-19 infection?

k.     CT severity score?
